# Spotlight on a Short-Time Treatment with the IL-4/IL-13 Receptor Blocker in Patients with CRSwNP: microRNAs Modulations and Preliminary Clinical Evidence

**DOI:** 10.3390/genes13122366

**Published:** 2022-12-15

**Authors:** Selena Mimmi, Nicola Lombardo, Domenico Maisano, Giovanna Piazzetta, Corrado Pelaia, Girolamo Pelaia, Marta Greco, Daniela Foti, Vincenzo Dattilo, Enrico Iaccino

**Affiliations:** 1 Department of Experimental and Clinical Medicine, University “Magna Græcia”, 88100 Catanzaro, Italy; 2 Otolaryngology Head and Neck Surgery, Department Medical and Surgical Sciences, University “Magna Græcia”, 88100 Catanzaro, Italy; 3 Dana-Farber Cancer Institute, Boston, MA 02215, USA; 4 Department of Health Sciences, University Magna Græcia of Catanzaro, 88100 Catanzaro, Italy

**Keywords:** miRNAs, antibody therapeutics, anti-IL-4/IL-13 receptor, dupilumab, nasal polyposis, interleukins, T cells

## Abstract

Already used for the treatment of some allergic and inflammatory diseases, such as asthma or atopic dermatitis, dupilumab has also been approved as add-on therapy for patients with CRSwNP, and it could represent the keystone to reducing the remission time as well as to improve healing and quality of life. On the other hand, the role of miRNAs as potential biomarkers of immune modulation is emerging. We analyzed the effects of a short-time treatment with dupilumab in patients with CRSwNP, analyzing the immune response modification as well as miRNAs modulations. First, in this early observation stage, all patients experienced remarkable improvement and were clinically stable. Indeed, we observed a significant decrease in CD4+ T cells and a significant reduction in total IgE (*p* < 0.05) and serum IL-8 levels (*p* < 0.01), indicating a reduction in the general inflammatory condition. In addition, we analyzed a panel of about 200 circulating miRNAs. After treatment, we noted a significant downregulation of hsa-mir-25-3p (*p*-value = 0.02415) and hsa-mir-185-5p (*p*-value = 0.04547), two miRNAs involved in the proliferation, inflammation, and dug-resistance, in accordance with the clinical status of patients. All these preliminary data aimed to identify new biomarkers of prognosis, identifiable with non-invasive procedures for patients. Further, these patients are still under observation, and others with different levels of responsiveness to treatment need to be enrolled to increase the statistical data.

## 1. Introduction

Chronic rhinosinusitis with nasal polyposis (CRSwNP) is an upper airway disorder, representing the most severe type of chronic rhinosinusitis, affecting 2–4% of the world population, more frequently people over 40 years old and with a higher incidence in men with respect to women [1]. Nasal polyps are inflammatory lesions growing in the nose or sinuses, and they can badly interfere with your quality of life and make sleeping difficult. Treatment includes nasal or oral medications, biologic injections, and surgery. However, these polyps may grow back [2].

CRSwNP is characterized by eosinophilic inflammation and T cell infiltration as well as by a type 2 inflammation planned by interleukin-4 (IL-4) and interleukin-13 (IL-13) [3]. Due to the negative affections on the quality of life of patients, the unknown treatment response, and the possible relapse of disease, additional studies are needed to explore the clinical and pathophysiological features of CRSwNP and identify novel biomarkers to improve the treatment and management of this disease. In this scenario, the introduction of a human monoclonal antibody that specifically targets and inhibits the activity of IL-4R and IL-13R, named dupilumab, is emerging as an innovative approach to improve the life quality in CRSwNP patients. [4]. In addition, microRNAs (miRNAs) modulate several physiological processes, such as immune system regulation or inflammatory condition [5], cancer [6], proliferation [7], and neurodegenerative disease [8]. miRNAs are short noncoding RNA molecules that bind the messenger RNA (mRNA), leading to the degradation and/or translational repression [9]. Thanks to their ability, they can modulate all biological mechanisms, so their deregulation could have a potential predictive role in diagnosis and prognosis. For this reason, miRNAs have gained consideration as reliable biomarkers in a wide range of pathological conditions, and their deregulation could significantly impact the prognosis of the disease and monitor the response to therapy [10].

In this study, we focused on evaluating the miRNAs modulation, inflammatory status, immune response, and molecular changes induced by dupilumab in our first enrolled group of CRSwNP patients.

## 2. Materials and Methods

### 2.1. Studies and Patients

Data were obtained from four randomized patients with diagnosis of CRSwNP, referring to the Respiratory Unit and to the Otolaryngology Unit of “Magna Græcia” University Hospital (Catanzaro, Italy). The diagnosis of CRSwNP was based on clinical criteria, nasal endoscopy and CT, as recommended by the European position paper on rhinosinusitis and nasal polyps [11]. Eligible patients were negative to COVID-19 swab and aged over 18 years with bilateral nasal polyposis. Patients were required to have bilateral endoscopic nasal polyp score (NPS) ≥5 (maximum score = 8) with ≥2 polyps per nostril and to exhibit ≥2 of the following criteria prior to screening: nasal obstruction or discharge, facial pain or pressure, or a reduced or lost sense of smell. Exclusion criteria included known coexisting clinical disease, such as severe asthma or atopic dermatitis, cystic fibrosis, immune deficiency, treatment with immunosuppressive therapy, or allergic bronchopulmonary aspergillosis.

Dupilumab was prescribed according to the existing eligibility indications and administered subcutaneously at an initial dose of 600 mg (two injections of 300 mg at different sites), followed by maintenance dosing of 300 mg every 2 weeks.

This study met the standards of Good Clinical Practice and the principles of the Declaration of Helsinki. All patients signed a written informed consent. The study was also carried out in agreement with a statement from the local Ethical Committee of Calabria Region (Catanzaro, Italy; document 182—20 May 2021).

### 2.2. Clinical Data

Pharmacodynamic measurements of total white blood cells count (WBC), neutrophils (NEUT), and eosinophils (EOS), and total serum IgE of patients were obtained from Clinical Pathology Units of University Magna Graecia of Catanzaro, under the supervision of Prof. Daniela Foti.

### 2.3. Samples Collection and Analysis

Peripheral blood samples were collected from all patients studied before starting the treatment (baseline T0) and after each injection (T1, T2, T3). Peripheral blood mononuclear cells (PBMCs) were isolated by Ficoll-PaqueTM Plus (Ge-Healthcare) according to manufacturer’s instructions. Isolated lymphocytes were stained with the combination of anti-CD3 FITC and anti-CD4 PE antibodies (Miltenyi Biotech, Bergisch Gladbach, Germany) and examined by flow cytometry (BD FACSCanto II). Serum sam, ples were analyzed using high sensitivity Biochip array technology (Randox Laboratories Ltd., Crumlin, County Antrim and Moorgate, London, England) according to manufacturer’s instructions for interleukins identification.

### 2.4. RNA Extraction and miRNAs Profiling

RNA extraction from serum samples referring to T0 (pre-treatment) and T3 (post-treatment) of each patient was performed using the miRNeasy Serum/Plasma kit (Qiagen, Valencia, CA, USA), following the manufacturer’s instructions. RNA from each sample was subjected to reverse transcription using the miRCURY LNATM RT Kit (Qiagen, Valencia, CA, USA) according to the manufacturer’s instructions. The levels of miRNAs in serum were assessed through Serum/Plasma Focus miRNA PCR Panel V5 (YAHS-106, Qiagen, Valencia, CA, USA), a panel comprising two 96-well PCR plates with 179 LNATM miRNAs primer sets and 5 spike-in control primer sets, focusing on serum- or plasma-relevant human miRNAs. Real-time PCR assays were performed using a Bio-Rad CFX96 Touch Real-Time PCR Detection System. The specificity of the PCR products was determined via melting curve analysis. The Ct values >36 were excluded from the analysis. Values below this threshold were considered for the analysis and calibrated by referring to the overall average of the UniSp3 inter-plate calibrator (IPC) Ct values, a spike-in present in triplicate in each plate. Results from the real-time PCR were normalized to the exogenous control cel-miR-39 and the fold expression was determined by the 2-ΔΔCt formula, where ΔΔCt corresponds to the difference between ΔCt value of each target miRNA in a patient at T3 and the geometric mean of the ΔCts for the same miRNA in the reference cohort T0. Heatmap with hierarchical cluster analysis (HCA) was carried out by using MeV software version 4.9.0 [12]. The clustering distance and clustering methods were “Euclidean” and “Complete linkage clustering”, respectively. Finally, for the statistical analysis, we considered only miRNAs that were detectable in at least three of the four patients.

### 2.5. Statistical Analyses

To assess variations over time of clinical and biochemical data, an ANOVA analysis for repeated measures, followed by Bonferroni’s multiple comparison test, was applied. Differences in serum miRNAs levels between two groups (T3 vs. T0) were analyzed by paired two-tailed *t*-test. *p*-values obtained for each individual miRNA were adjusted for multiple testing by Benjamini–Hochberg method [13,14]. The analyses were conducted using GraphPad Prism software (San Diego, CA, USA), and differences were considered significant at * *p* ≤ 0.05, ** *p* ≤ 0.01, and *** *p* ≤ 0.001.

### 2.6. Pathway Analysis

In order to identify the biological processes in which the investigated miRNAs are involved, a pathway analysis was conducted by using the freely available online software mirPath v.3 (http://www.microrna.gr/miRPathv3; accessed on 30 June 2022). This tool is able to identify pathways regulated by specific miRNAs by utilizing predicted interactions between miRNAs and target genes (DIANA-microT-CDS algorithm) or experimentally validated miRNA interactions (DIANA-TarBase). The analysis output consists of functional annotations for one or more miRNAs at the same time, referring all analyses to the Kyoto Encyclopedia of Genes and Genomes (KEGG) molecular pathways.

## 3. Results

**Figure 1:**
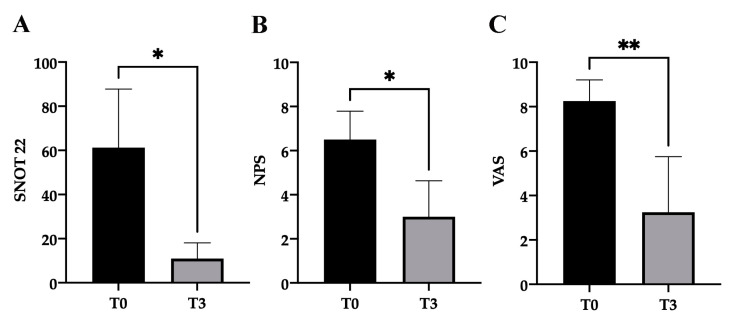
Clinical parameters pre (T0) and post (T3) treatment with Dupilumab. Patients were evaluated with regards to canonical diagnostic/prognostic scores, such as SNOT-22 (panel **A**), NPS (panel **B**), and VAS (panel **C**). All parameters were expressed as means ± SD. Differences between two groups (T3 vs. T0) were analyzed by paired two-tailed *t*-test; * *p* < 0.05, ** *p* < 0.01.

To verify the putative clinical effects of the treatment with dupilumab in patients with CRSwNP, we firstly assessed specific diagnostic/prognostic scores such as SNOT-22 [15], NPS [16], and VAS [17]. All four patients showed a significant amelioration over time for all the evaluated scores (F = 11.74 and *p* = 0.0018 for SNOT-22, F = 6.11 and *p* = 0.0149 for NPS, F = 9.46 and *p* = 0.0038 for VAS), with a more pronounced reduction at T3 compared to baseline (*p* < 0.01 for SNOT-22, *p* < 0.05 for NPS and VAS) (Table 1 and Figure 1).

**Figure 2:**
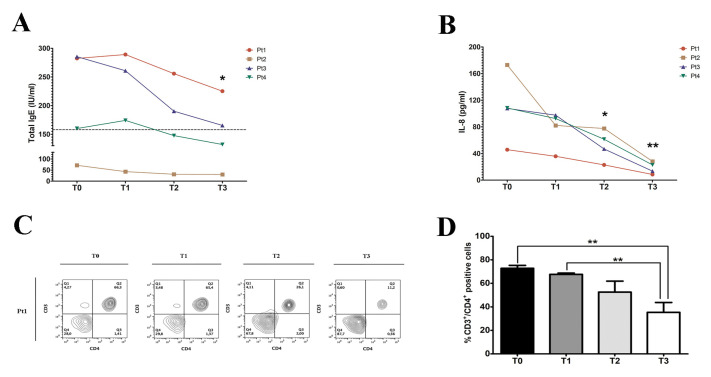
IgE (**A**) and IL-8 (**B**) levels in serum of patients with CRSwNP before (T0) and after (T1, T2, T3) treatment with Dupilumab; dotted line indicates the reference value threshold. (**C**) Flow cytometry assay of circulating CD3^+^/CD4^+^ T cells in blood of the most representative patient (Pt1) during the observation; the percentage of CD3^+^/CD4^+^ T cells was reported in the Q2 square of each plot panel. (**D**) Percentage of CD3^+^/CD4^+^ T cells in the overall patients at the indicated timepoints; data are reported as the mean ± s.e.m. Data were analyzed by ANOVA for repeated measures, followed by Bonferroni’s multiple comparison test; * *p* < 0.05, ** *p* < 0.01.

At the same time, we evaluated the variation of total IgE concentrations. We observed a significant reduction in serum IgE levels during the treatment (T1, T2, T3) in all patients (F = 7.51 and *p* = 0.008), which became more evident at the end of the treatment (T3) compared to baseline levels (*p* < 0.05) (Figure 2A). Moreover, we observed intriguing variations in the IL-8 levels over time (F = 12.43 *p* = 0.0015) (Figure 2B), with a significant reduction at the end of treatment compared to baseline (*p* < 0.01), in accordance with the reduction in IgE levels. By flow cytometry, we observed a reduction in circulating CD4+ T cells over time in all treated patients (F = 12.71 and *p* = 0.0014), especially at T3 compared to baseline (*p* < 0.01) (Figure 2C,D).

These findings suggest a lowering of the CD4- mediated inflammatory3 response, which correlates with the clinical course of the patients. Indeed, no inflammatory processes were found, and all patients were clinically stable at T3.

**Figure 3:**
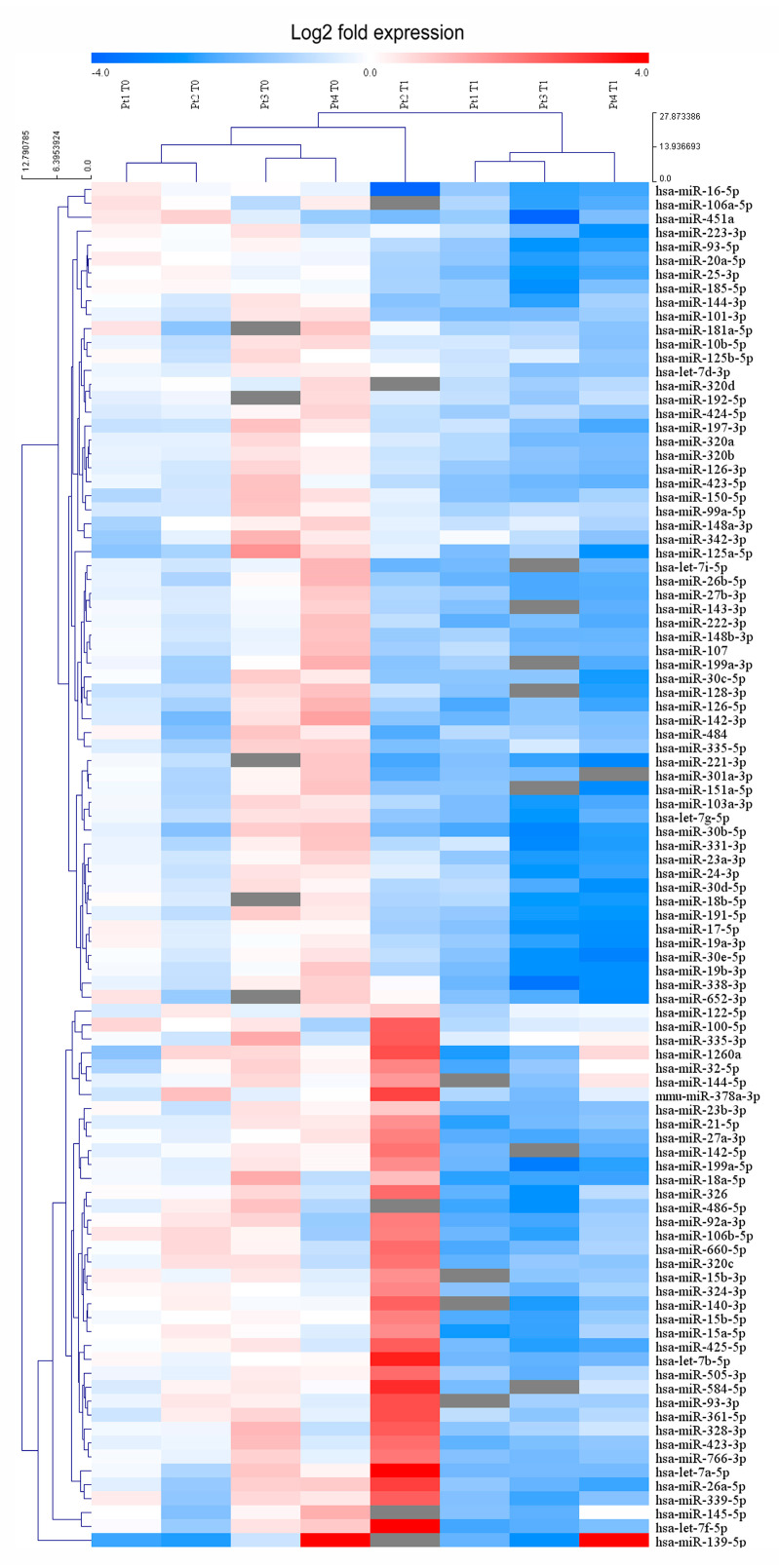
Serum miRNAs profiling of patients at baseline (T0) and after treatment (T3). Heatmap displaying the results of a hierarchical cluster analysis (HCA) conducted on the patients’ serum levels of a 98 miRNAs panel at baseline and after the end of the treatment. Log2 fold expression values of reference are reported in the upper bars.

Finally, we analyzed possible modifications in circulating miRNAs. To accomplish this aim, for each patient, we performed a serum miRNAs profiling at baseline and at the end of the treatment. Of the 179 miRNAs screened, 98 were detectable in at least 3 of the 4 patients and were then considered in subsequent statistical analyses (Figure 3).

**Figure 4:**
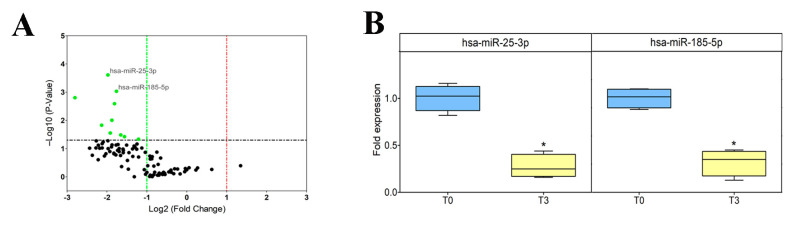
(**A**) Volcano plot of the 98 serum miRNAs analyzed by qRT-PCR (T3 vs. T0). Significant down-regulated miRNAs are localized in the upper-left section (dots colored green). (**B**) Serum miR-25-3p and miR-185-5p levels at T3 compared to baseline. Values are represented in whiskers plots as fold expression. Statistical significance is indicated at the top of the graph. * *p* ≤ 0.05.

The analysis revealed a significant and consistent (≥2-fold change) deregulation for ten microRNAs after the treatment and suggestive but not significant deregulation for other two miRNAs (Figure 4A).

Interestingly, some of these miRNAs belong to common genomic clusters, suggesting that the treatment could act on specific molecular hubs in modulating miRNA levels. Afterward, to reduce the false discovery rate (FDR), we applied the Benjamini–Hochberg procedure that confirmed a significant downregulation only for hsa-miR-25-3p and hsa-miR-185-5p (Figure 4B) with a fold change of 0.25 and 0.29, respectively (Table 2).

Furthermore, the pathway analysis conducted on the two miRNAs modulated after the treatment showed that 34 KEGG pathways were significantly (*p* < 0.05) enriched with genes representing targets of these miRNAs (Table 3).

Pathway analysis revealed a crucial role of these miRNAs in the regulation of target genes involved in several molecular mechanisms regulating inflammation and immune response, as well as underlying cancer pathogenesis, all processes that are known to affect the pathophysiology of the disorder. In accordance with the pathway analysis, literature data report that the principal targets of hsa-miR-25-3p are two tumor suppressor genes involved in the proliferation, apoptosis, and DNA repair (BTG2) [18], and in the PI3K/AKT pathway (PTEN) [19]. Differently, hsa-miR-185-5p seems to be implicated in drug resistance [20] and in T cell regulation by modulating PD-L1 concentration [21]. Possible CRSwNP-related pathways in which the identified miRNAs are involved were graphically illustrated in Supplementary Figure S1.

## 4. Discussion

CRSwNP is an inflammatory disease that causes severe implications for respiration, characterized by drug resistance, and, thus, negatively impacting the quality of life. This makes the identification of new targeted therapies and specific noninvasive biomarkers an urgent need. In this study, we analyzed a preliminary group of four patients with the diagnosis of CRSwNP and we evaluated the response to dupilumab treatment. Dupilumab is a monoclonal antibody with the ability to bind and block the IL-4/IL-13 receptor, already approved for the treatment of atopic dermatitis. Recently it was approved as add-on therapy to intranasal corticosteroids for the treatment of adult patients with severe CRSwNP when systemic corticosteroid therapy and/or surgery have not adequately controlled the disease. The European approval is based on two pivotal Phase III clinical trials (24-week SINUS-24 and 52-week SINUS-52) evaluating treatment with dupilumab as an adjunct to standard of care intranasal corticosteroids, versus placebo plus intranasal corticosteroids [22]. Here we have analyzed the inflammatory modulations due to dupilumab treatment, as well as modulation of miRNAs expression, to identify new prognostic biomarkers. It was well demonstrated that increased IgE and IL-8 concentrations may be found in patients with CRSwNP stimulating multiple inflammatory pathways [23,24]. Indeed, IL-8 plays a crucial role in the inflammatory processes acting as a chemotactic factor for neutrophils and lymphocytes, as angiogenic factor, as well as tumor progression [25,26,27], and an IL-8-dominant inflammatory profile is frequently found in patients with CRSwNP [28,29]. Since patients showed a clear improvement in both clinical stage and quality of life after dupilumab treatment accompanied by a reduction in IL-8 concentration, this evidence confirms, for the first time, the downstream action of dupilumab on IgE and IL-8, which led to the amelioration of the inflammatory status and to counteract the CRSwNP progression. Furthermore, several studies pointed out the key role played by circulation CD4 positive T cells in the context of inflammation [30,31,32]. Our data demonstrated that after treatment, circulating CD4 positive T cells decreased over time, lowering inflammatory levels.

In recent decades, the role of miRNAs as biomarkers of disease progression is creased [33,34,35,36]. miRNAs are crucial post-transcriptional modulators acting as inhibitors of gene expression, resulting in the modulation of all biological pathways and functions [37,38]. In addition, the role of specific miRNAs in the pathogenesis of asthma and airway hyperresponsiveness was well documented, as well as the polarization of adaptive immune responses, activation of T cells [39], regulation of eosinophil development [40], and modulation of cytokine-driven responses [41]. Therefore, we asked whether also in CRSwNP they could be helpful for a rapid noninvasive prognosis. Analyzing a panel of ~200 miRNAs, we found the downregulation of hsa-miR-25-3p and hsa-miR-185-5p. Pathway analysis and literature data indicate that hsa-miR-25-3p and hsa-miR-185-5p are involved in inflammatory modulation [18], drug-resistance [20], and T cell regulation [21], all process implicated also in CRSwNP pathogenesis.

Patients with CRSwNP are subjected to very invasive diagnostic and prognostic practices, often not well accepted, especially in the most fragile patients. For this reason, there is a need to identify new prognostic biomarkers that can be evaluated with less invasive methods, such as a simple blood draw. In this contest, if validated in a large cohort of patients, hsa-miR- 25-3p and hsa-miR-185-5p can be considered in clinical practice as new biomarkers in CRSwNP prognosis, using noninvasive approach.

## 5. Conclusions

Therefore, according to the literature, the downregulation of hsa-miR-25-3p and hsa-miR-185-5p is in accordance with the molecular results we found and with the clinical status of patients. Taken together, these data represent preliminary evidence of dupilumab treatment efficacy in the course of CRSwNP, resulting in an impressive impact on patients’ quality of life. Indeed, dupilumab was helpful in improving the clinical conditions in treated patients, lowering the values of the main actors of the inflammation responsible for the pathogenesis and progression of CRSwNP. We are aware that our observation group expects a small number of patients (n.4), but our preliminary data open the way to identify potential new biomarkers of prognosis and treatment efficacy, using noninvasive approach with respect to the conventional clinical procedures.

## Figures and Tables

**Table 1:** Clinical and biochemical data of enrolled patients with CRSwNP diagnosis. Symbol (*) indicates outlier values. Values of white blood cells (WBC), neutrophils (NEUT), and eosinophils (EOS) were obtained from blood count reports.

Patients	Analytes	T0	T1	T2	T3	Reference Value
Patient 1 46 y male	Total IgE WBC NEUT EOS SNOT-22 NPS VAS	282.5 * 6.61 3.43 0.49 84 5 8	289.2 * 5.84 2.99 0.18 64 3 4	255.8 * 5.99 3.87 1.54 * 25 1 6	225.2 * 6.76 2.23 1.99 * 19 1 6	<158 (IU/mL) 5.2–12.4 (×10^3^/uL) 0–7 (×10^3^/uL) 0–0.8 (×10^3^/uL) [15] [16] [17]
Patient 2 60 y female	Total IgE WBC NEUT EOS SNOT-22 NPS VAS	71.4 7.58 4.21 0.82 * 43 8 9	43.1 7.86 4.55 0.57 8 7 1	31.2 9.18 5.65 0.04 1 2 0	30.2 7.65 4.22 0.56 2 3 0	<158 (IU/mL) 5.2–12.4 (×10^3^/uL) 0–7 (×10^3^/uL) 0–0.8 (×10^3^/uL) [15] [16] [17]
Patient 3 30 y female	Total IgE WBC NEUT EOS SNOT-22 NPS VAS	285.5 * 10.65 6.20 1.04 * 84 6 9	260.9 * 9.45 4.25 2.77 * 26 5 3	190.3 * 11.32 4.13 5.12 * 38 6 5	165.4 * 13.37 * 5.27 4.49 * 13 3 4	<158 (IU/mL) 5.2–12.4 (×10^3^/uL) 0–7 (×10^3^/uL) 0–0.8 (×10^3^/uL) [15] [16] [17]
Patient 4 51 y male	Total IgE WBC NEUT EOS SNOT-22 NPS VAS	160.0 * 7.05 3.81 0.31 34 7 8	174.2 * 7.61 4.43 0.4 17 7 4	147.6 * 7.49 4.31 0.27 12 6 6	132.1 7.44 4.23 0.37 10 5 6	<158 (IU/mL) 5.2–12.4 (×10^3^/uL) 0–7 (×10^3^/uL) 0–0.8 (×10^3^/uL) [15] [16] [17]

**Table 2:** Serum miRNAs differentially expressed in patients after treatment (T3) compared to baselines (T0). Bold characters indicate significant *p*-values both at a nominal level and adjusted for multiple testing. Differences between two groups (T3 vs. T0) were analyzed by paired two-tailed *t*-test, followed by Benjamini–Hochberg correction.

miRNA ID	miRBase Accession Number	Cluster (<10 Kb)	Genomic Coordinates (GRCh38)	Fold Expression (T3 vs. T0)	Nominal *p*-Value	Adjusted *p*-Value
hsa-miR-93-5p hsa-miR-25-3p	MIMAT0000093 MIMAT0000081	#1	chr7: 100093768-100093847 [-] chr7: 100093560-100093643 [-]	0.27 ± 0.09 0.25 ± 0.09	**0.00991** **0.00024**	0.19429 **0.02415**
hsa-miR-185-5p	MIMAT0000455		chr22: 20033139-20033220 [+]	0.29 ± 0.07	**0.00092**	**0.04547**
hsa-miR-16-5p	MIMAT0000069		chr13: 50048973-50049061 [-]	0.13 ± 0.07	**0.00156**	0.05123
hsa-miR-17-5p hsa-miR-19a-3p hsa-miR-20a-5p	MIMAT0000070 MIMAT0000073 MIMAT0000075	#2	chr13: 91350605-91350688 [+] chr13: 91350891-91350972 [+] chr13: 91351065-91351135 [+]	0.22 ± 0.07 0.26 ± 0.09 0.28 ± 0.06	**0.01479** **0.02802** **0.00256**	0.22480 0.30224 0.06296
hsa-miR-144-3p hsa-miR-451a	MIMAT0000436 MIMAT0001631	#3	chr17: 28861533-28861618 [-] chr17: 28861369-28861440 [-]	0.32 ± 0.06 0.21 ± 0.07	**0.03275** 0.06715	0.30218 0.30224
hsa-miR-101-3p	MIMAT0000099		chr1: 65058434-65058508 [-]	0.34 ± 0.03	**0.03785**	0.30221
hsa-miR-143-3p hsa-miR-145-5p	MIMAT0000435 MIMAT0000437	#4	chr5: 149428918-149429023 [+] chr5: 149430646-149430733 [+]	0.24 ± 0.06 0.42 ± 0.20	0.05311 **0.04915**	0.30223 0.30219

**Table 3:** Pathway analysis showing the biological processes in which the investigated miRNAs are involved. This table show the pathway affected by hsa-miR-25-3p and hsa-miR-185-5p, the measure of the significance (*p*-value column), the number of the regulated genes belonging to the pathway (# genes column) and if both the miRNAs are implicated in the modulation of the pathway or not (# miRNAs column).

#	KEGG Pathway	*p*-Value	# Genes	# miRNAs
1	Prion diseases (hsa05020)	5.70830884354 × 10^−19^	4	2
2	Hippo signaling pathway (hsa04390)	1.15396059352 × 10^−9^	30	2
3	Adherens junction (hsa04520)	8.956826783 × 10^−9^	20	2
4	Lysine degradation (hsa00310)	1.59930615032 × 10^−7^	12	2
5	Cell cycle (hsa04110)	1.59930615032 × 10^−7^	37	2
6	ECM-receptor interaction (hsa04512)	3.3044921882 × 10^−7^	15	2
7	Viral carcinogenesis (hsa05203)	1.78296527476 × 10^−6^	42	2
8	Proteoglycans in cancer (hsa05205)	8.42687201939 × 10^−6^	37	2
9	Valine, leucine and isoleucine biosynthesis (hsa00290)	3.3044921882 × 10^−7^	2	1
10	Protein processing in endoplasmic reticulum (hsa04141)	0.000981175574837	30	2
11	Thyroid hormone signaling pathway (hsa04919)	0.00240597308507	23	2
12	Chronic myeloid leukemia (hsa05220)	0.00589932113114	18	2
13	Estrogen signaling pathway (hsa04915)	0.00787690932391	17	2
14	FoxO signaling pathway (hsa04068)	0.00923087959942	28	2
15	Endometrial cancer (hsa05213)	0.0105650837333	12	2
16	p53 signaling pathway (hsa04115)	0.0106079452352	16	2
17	Renal cell carcinoma (hsa05211)	0.0106190669021	15	2
18	Bladder cancer (hsa05219)	0.0106190669021	12	2
19	MAPK signaling pathway (hsa04010)	0.0172375957743	37	2
20	Oocyte meiosis (hsa04114)	0.0172375957743	22	2
21	Dorso-ventral axis formation (hsa04320)	0.0172375957743	9	2
22	Pathways in cancer (hsa05200)	0.0172375957743	52	2
23	Colorectal cancer (hsa05210)	0.0172375957743	12	2
24	Prostate cancer (hsa05215)	0.0172375957743	19	2
25	Regulation of actin cytoskeleton (hsa04810)	0.0189043683549	34	2
26	Thyroid cancer (hsa05216)	0.0201059248122	7	2
27	Focal adhesion (hsa04510)	0.0233501267056	36	2
28	TGF-β signaling pathway (hsa04350)	0.0242934910873	13	2
29	Platelet activation (hsa04611)	0.0242934910873	23	2
30	Melanoma (hsa05218)	0.0242934910873	13	2
31	Hepatitis B (hsa05161)	0.0286316028687	27	2
32	2-Oxocarboxylic acid metabolism (hsa01210)	0.0331222256653	4	1
33	DNA replication (hsa03030)	0.0353568029332	8	2
34	Glioma (hsa05214)	0.0353568029332	13	2

## Data Availability

Data is contained within the article and Supplementary Material.

## Supplementary Materials

The following supporting information can be downloaded at: https://www.mdpi.com/article/10.3390/genes13122366/s1, Figure S1: More representative pathway in which the identified miRNAs are involved, according to the mirpath3 database.
