# Supplementary material for: Spotlight on a Short-Time Treatment with the IL-4/IL-13 Receptor Blocker in Patients with CRSwNP: microRNAs Modulations and Preliminary Clinical Evidence
Source: Genes (Basel). 2022 Dec 15;13(12):2366. doi: 10.3390/genes13122366 (PMC9777725; doi:10.3390/genes13122366)
Supplement: Supplementary file 1 [file genes-13-02366-s001.zip › genes-2104481-supplementary.pdf]

Supplementary Figure S1: More representative pathway in which the identified miRNAs are involved, according to the mirpath3 database.

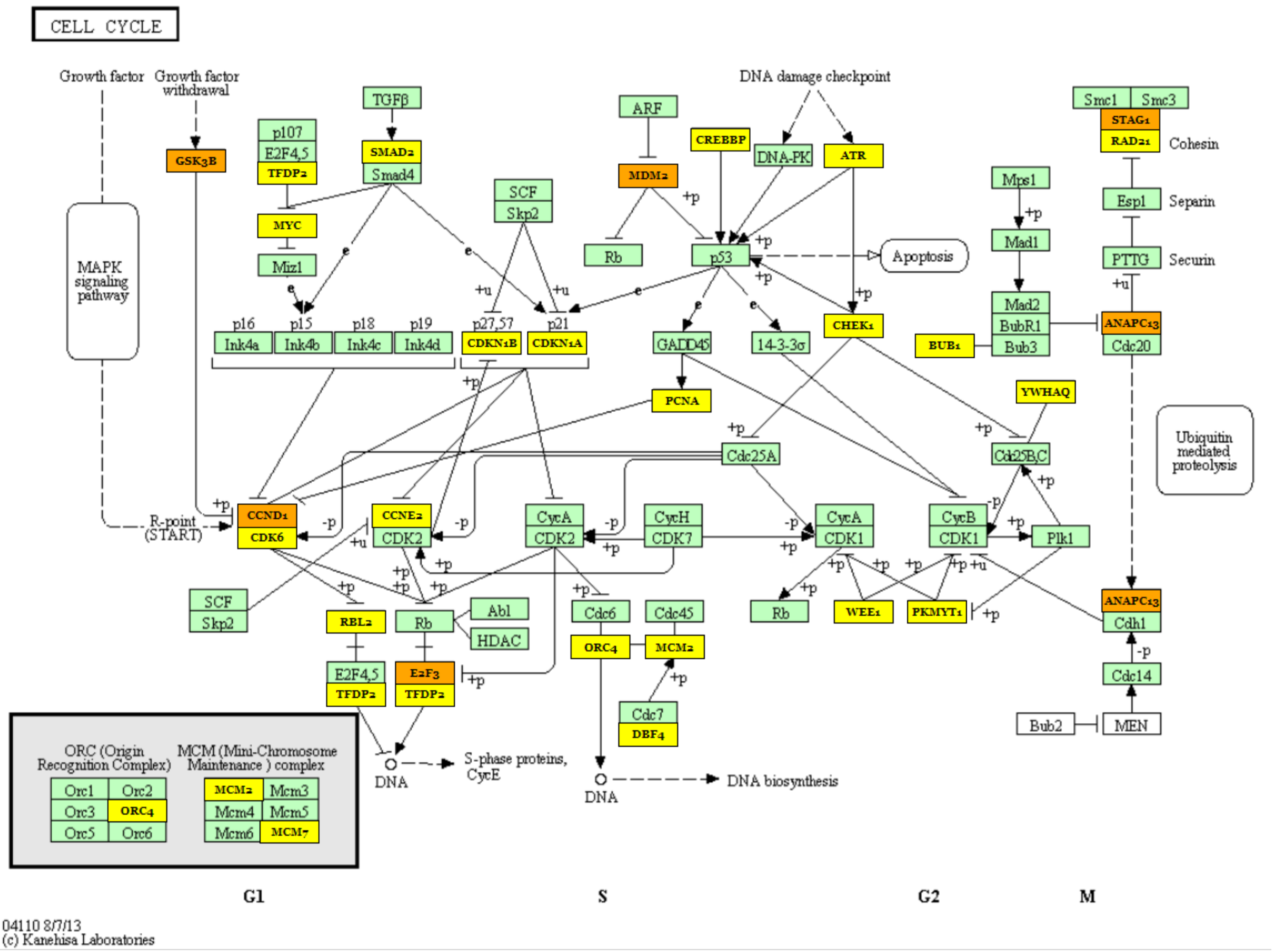

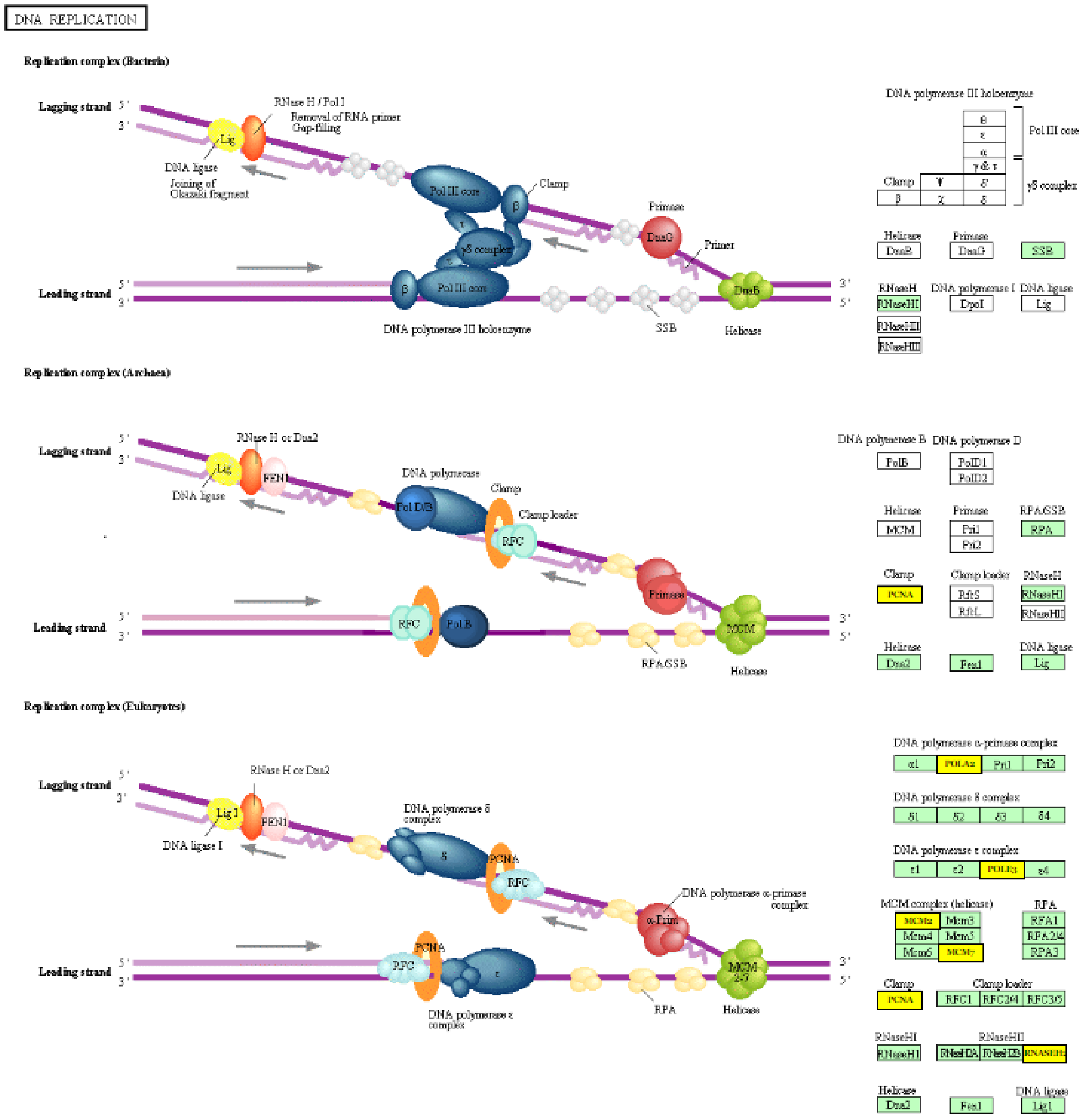

03030 1/23/15
(c) Kanehisa Laboratories

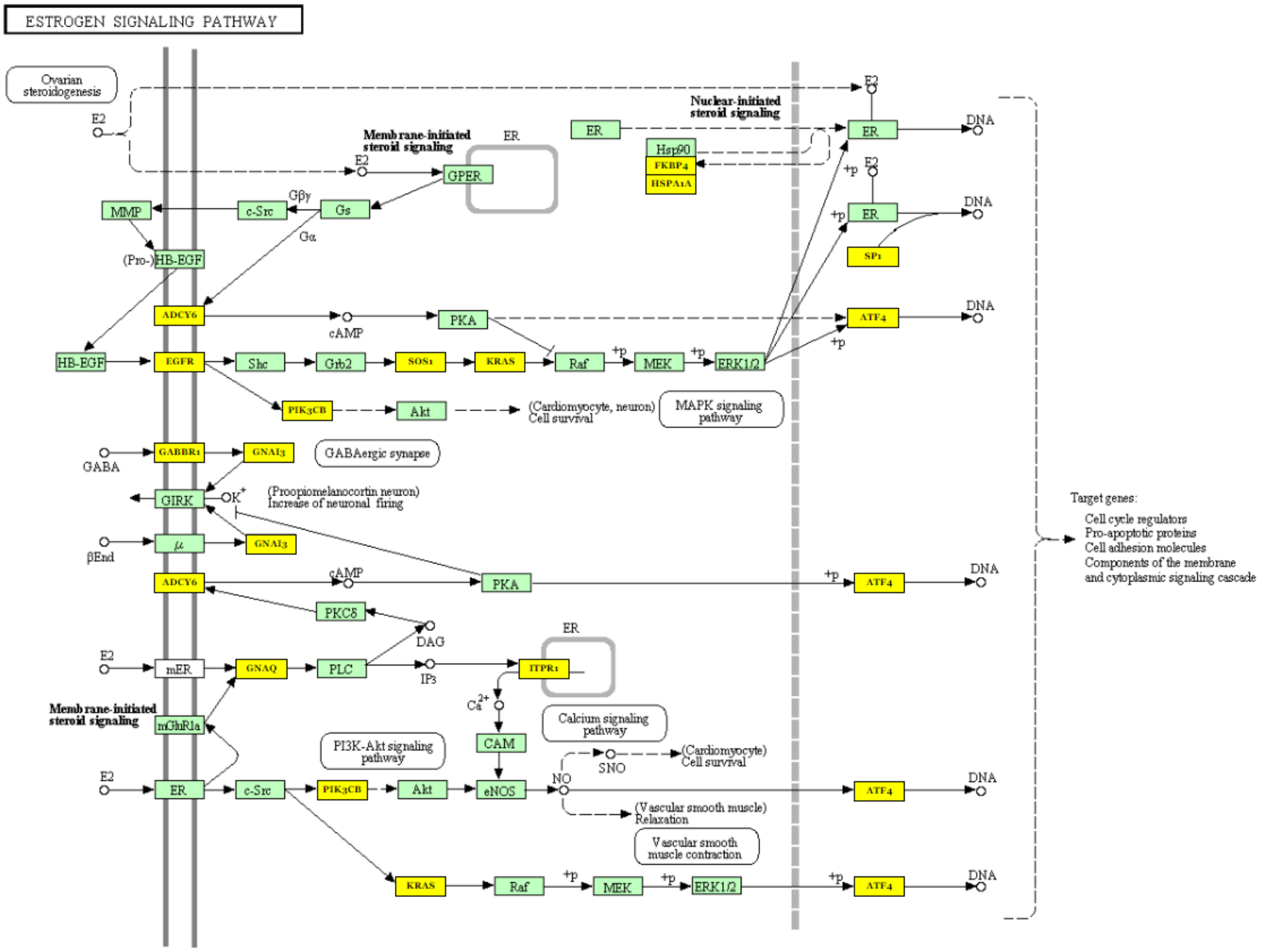

04915 2/6/15
(c) Kanehisa Laboratories

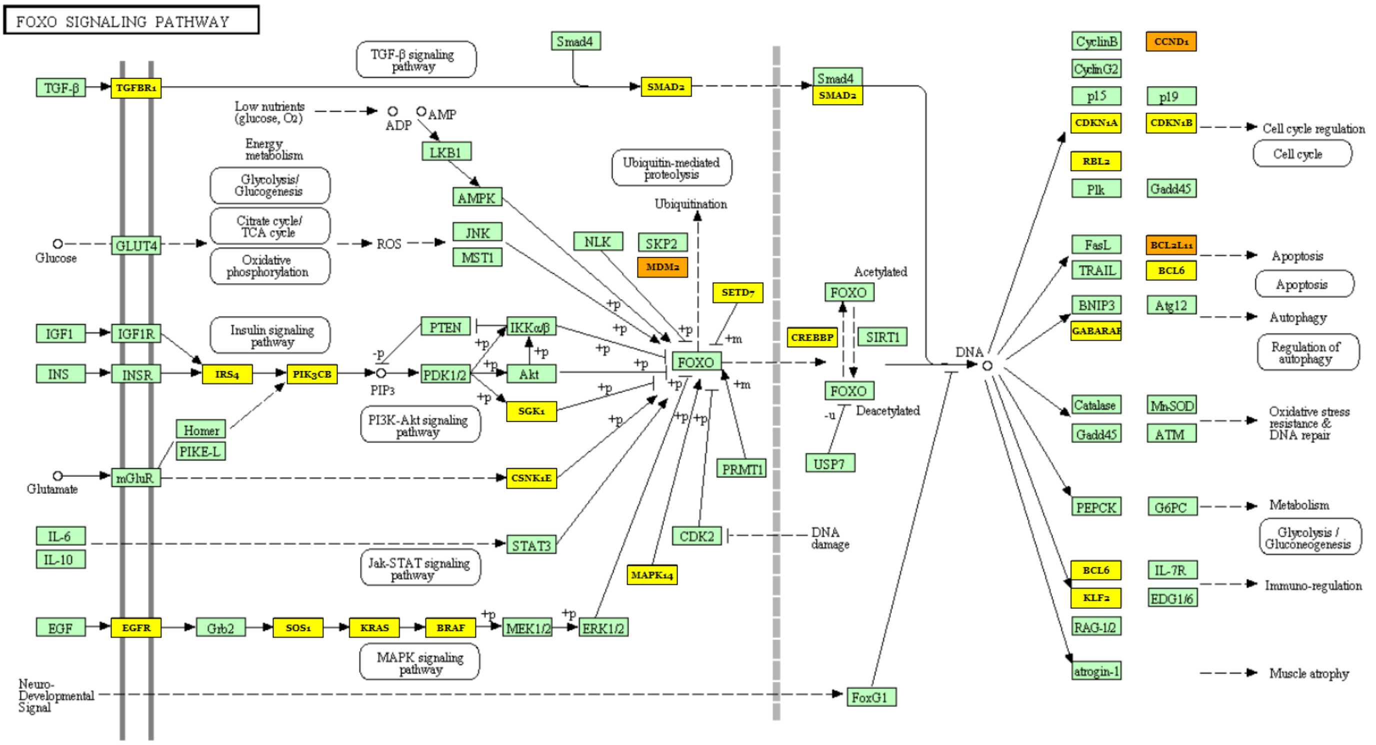

04068 5/8/14
(c) Kanehisa Laboratories

: gene contained in 1 list

: gene contained in > 1 lists

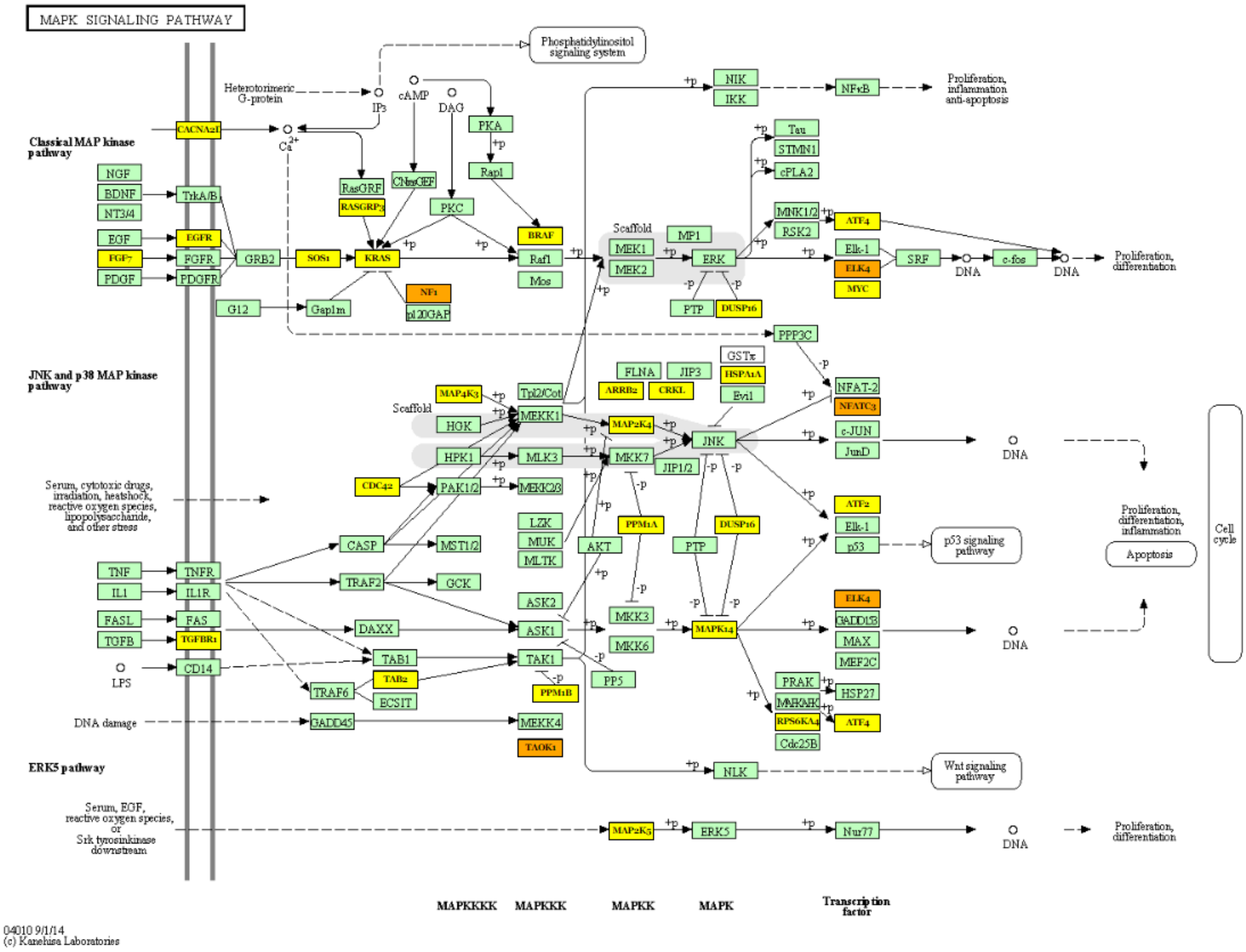

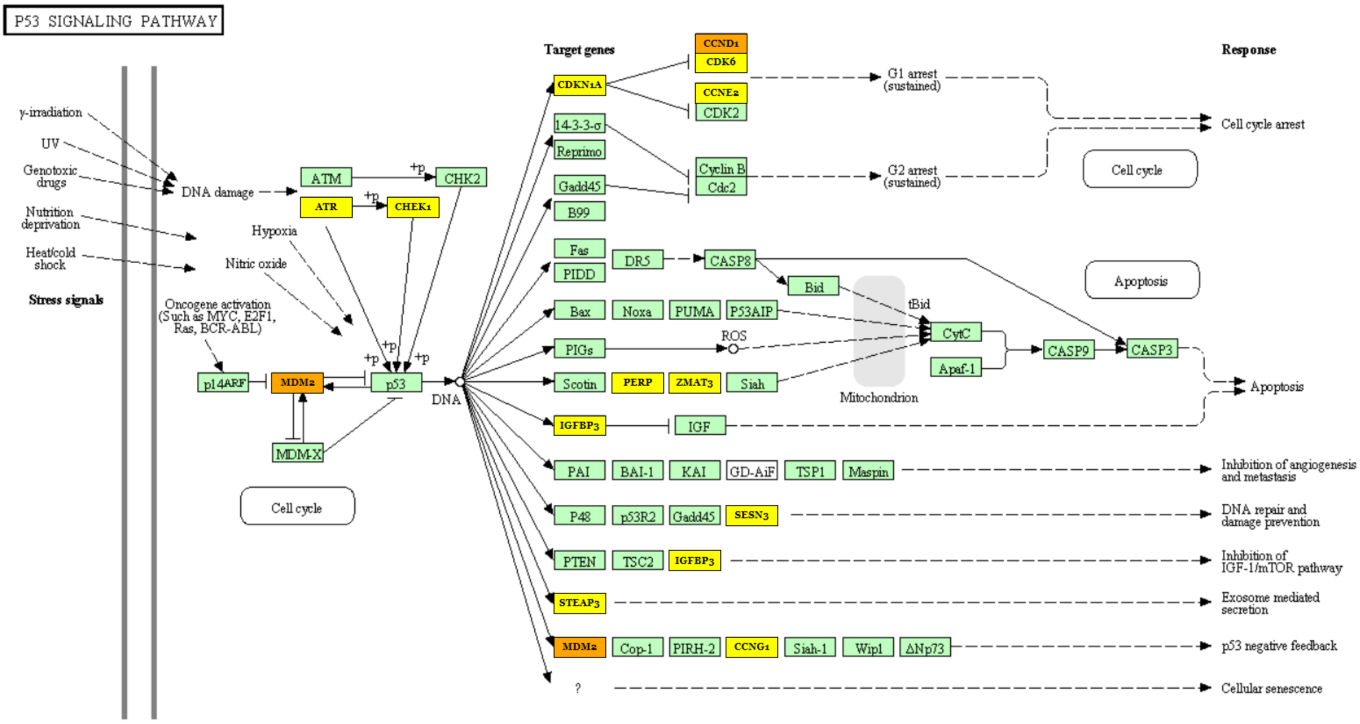

04115 3/7/13
(c) Kanehisa Laboratories

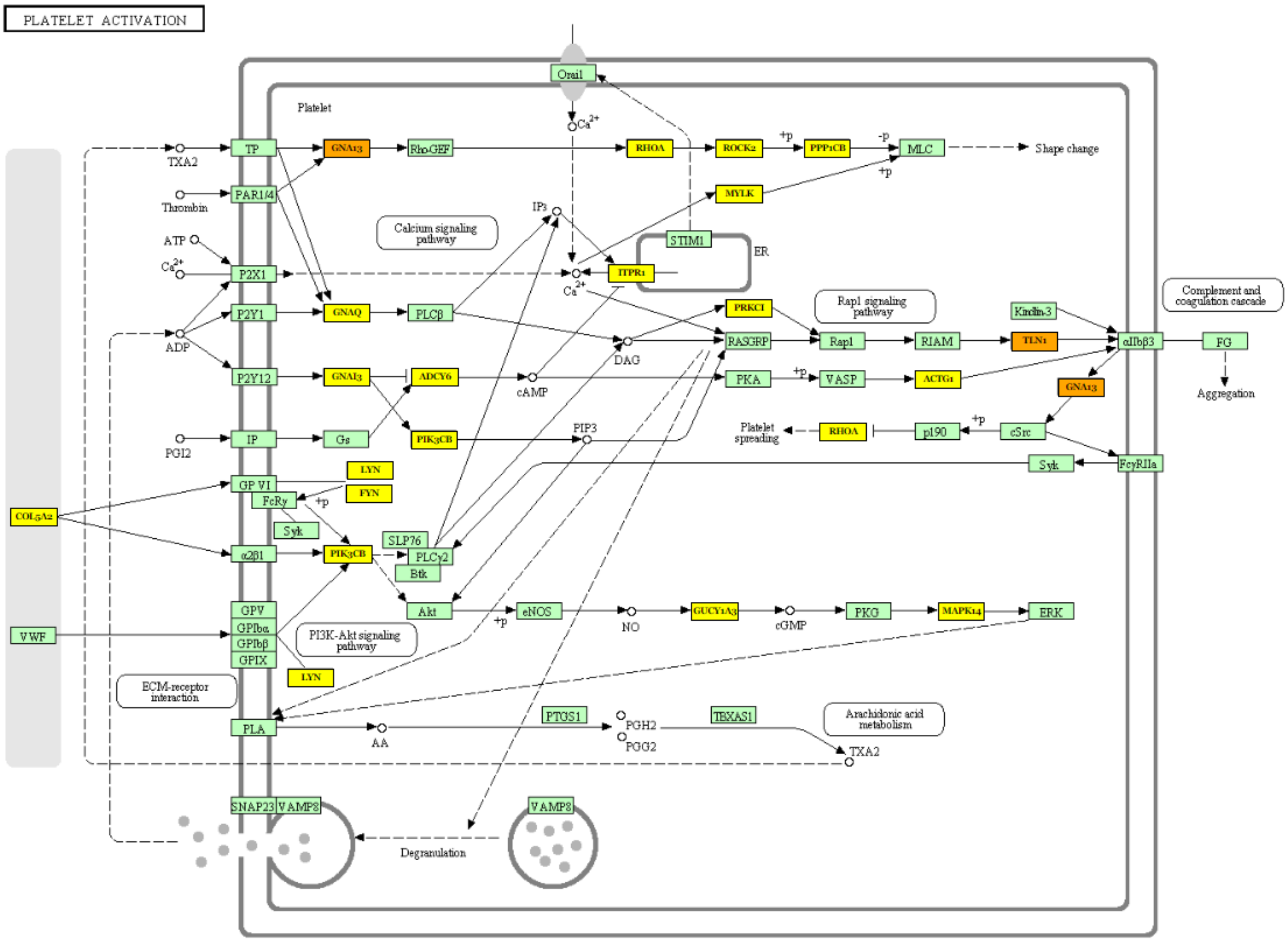

04611 2/13/15
(c) Kanehisa Laboratories

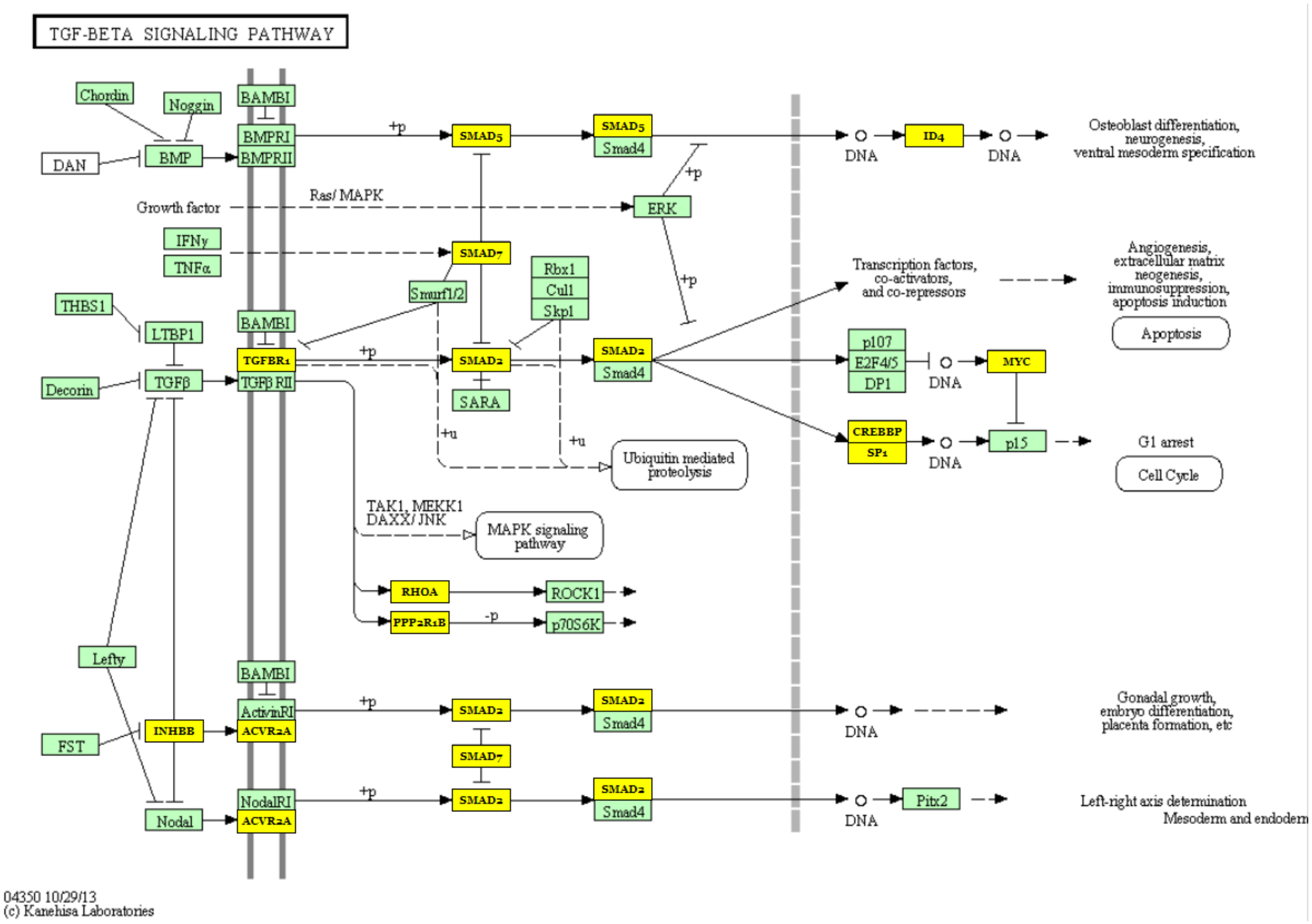

TGF-BETA SIGNALING PATHWAY
Chordin
Noggin
DAN
BMP
BAMBI
BMPRI
BMPRII
+p
SMAD5
SMAD5
Smad4
ID4
DNA
DNA
Osteoblast differentiation, neurogenesis, ventral mesoderm specification
Growth factor
Ras/ MAPK
ERK
+p
IFNγ
TNFα
SMAD7
Rbx1
Cul1
Skp1
Smurf1/2
Transcription factors, co-activators, and co-repressors
Angiogenesis, extracellular matrix neogenesis, immunosuppression, apoptosis induction
Apoptosis
THBS1
LTBP1
BAMBI
TGFBR1
TGFβ RII
SMAD2
SMAD2
Smad4
SARA
p107
E2F4/5
DP1
DNA
MYC
Decorin
TGFβ
CREBBP
SP1
DNA
p15
G1 arrest
Cell Cycle
+u
+u
Ubiquitin mediated proteolysis
TAK1, MEKK1 DAXX/ JNK
MAPK signaling pathway
RHOA
ROCK1
PPP2R1B
-p
p70S6K
Lefty
BAMBI
ActivinRI
ACVR2A
SMAD2
SMAD2
Smad4
DNA
Gonadal growth, embryo differentiation, placenta formation, etc
FST
INHBB
SMAD7
Nodal
NodalRI
ACVR2A
SMAD2
SMAD2
Smad4
DNA
Pitx2
Left-right axis determination
Mesoderm and endoderm
04350 10/29/13
(c) Kanehisa Laboratories

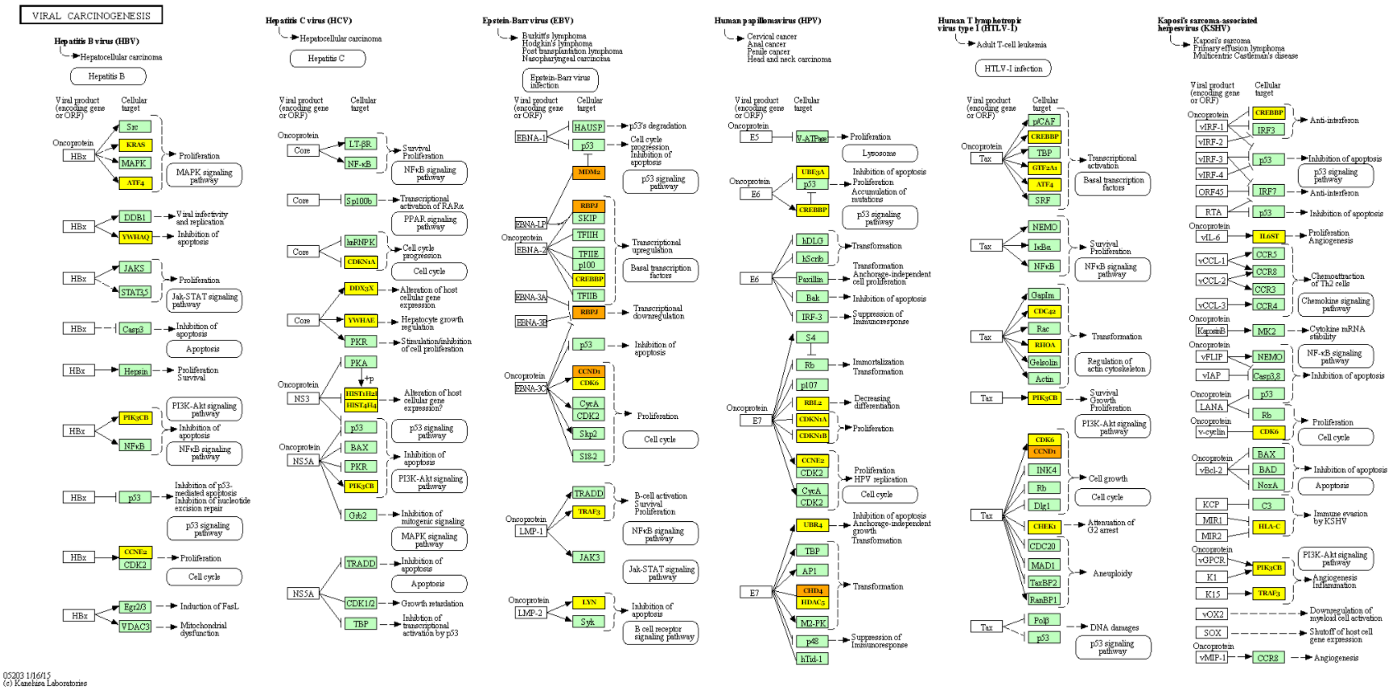

VIRAL CARCINOGENESIS
Hepatitis B virus (HBV)
Hepatitis C virus (HCV)
Epstein-Barr virus (EBV)
Human papillomavirus (HPV)
Human T lymphotropic virus type I (HTLV-I)
Kaposi's sarcoma-associated herpesvirus (KSHV)
05203 1/16/15
(c) Kanehisa Laboratories
